# To enhance the quality of CPR performed by youth layman

**DOI:** 10.1186/s12245-019-0247-6

**Published:** 2019-10-07

**Authors:** Anna Abelsson, Annette Nygårdh

**Affiliations:** School of Health Sciences, Jönköping University, PO Box 1026, 551 11 Jönköping, Sweden

**Keywords:** CPR, Intervention, Layman, Simulation

## Abstract

**Background:**

By educating laymen, survival after cardiac arrest can increase in society. It is difficult to reach the entire population with cardiopulmonary resuscitation (CPR) training. However, if 15% of the population knows how to perform CPR, an increase in short- and long-term survival in patients suffering a cardiac arrest could be seen. To educate youth is a way to reach parts of the population. This study aimed to investigate the effect of a 2-h CPR intervention for youth.

**Methods:**

Data were collected through an intervention utilizing simulation and consisted of a pre- and post-assessment of 50 participants’ CPR performance.

**Results:**

The participants’ compression depths are, after training, within guidelines. However, the compression rate increases from within limits (117) to become too fast (128). The range of the minimum compression rate rises from 70 to 92 which is an improvement. The ventilation volume increases from 112 ml in pre-test to 579 ml in post-test. In the pre-test, 88% of the participants did not succeed in securing an open airway; only six participants succeeded in securing an open airway. In the post-test, 49% of the participants underperform in the ventilation. However, only 12 participants failed in securing an open airway in the post-test. Compression recoil and hand position marginally improved from pre- to post-test.

**Conclusion:**

Educating young people at school is one way to disseminate CPR knowledge in society. In this study, the ventilation of the patient arose as a major weakness. To be able to establish an open airway and ventilate the patient with the correct volume as well as to overcome the psychological barrier to initiate mouth-to-mouth ventilation seems to require more than 2 h training. The training may need to consist of repeated sessions over the year with feedback, to give young people the skills to perform CPR with good quality.

## Background

Immediate start of CPR improves survival after cardiac arrest as the cerebral neurodegeneration starts already after 3–5 min [1]. Correctly performed CPR consists of correct chest compressions, containing adherence to rate, depth, full recoil, and fraction. The quality of the CPR affects the patient’s short-term and long-term survival at a cardiac arrest [2]. By educating laymen, survival after cardiac arrest can increase in society. It is difficult to reach the entire population when CPR training is not obligatory. However, if 15% of the population can perform CPR, one could see a statistically significant increase in CPR results [3].

In 2015, a joint statement was published from the European Resuscitation Council (ERC), the European Patient Safety Foundation (EPSF), the International Liaisons Committee on Resuscitation (ILCOR), the World Federation of Societies of Anesthesiologists (WFSA), and the World Health Organization (WHO), endorsing the implementation of “Kids Save Lives” project. The project recommends a 2-h annual CPR training for schoolchildren starting at the age of 12 years, which is the optimal age to start teaching children cardiac compressions [3]. Previous research has shown that first aid and CPR education significantly increase the basic life support knowledge of schoolchildren to assist in an emergency situation [4]. Continuous training can result in a sustainable acquisition and maintenance of CPR performance by schoolchildren [5]. First aid and CPR are part of handling emergencies and are vital knowledge of all citizens, from young children, adolescents, and adults. Knowledge can contribute to reduced suffering, reduced risk of injury, and increased chance of survival in the event of illness or accident [6].

A detailed curriculum has been developed for CPR education in Swedish schools. Schoolchildren receive first aid and CPR training from elementary school to high school, 5–15 years old [6, 7]. In elementary school, 5–9 years, students are trained on how to check if the person is conscious or unconscious, open the airway, do mouth-to-mouth ventilation, put the person in recovery position, and call the emergency number. Aged 10–12 years, the students are trained in what to do in a choking emergency (5 back blows and 5 abdominal thrusts), mouth-to-mouth ventilation, and chest compressions. Other parts to train are wounds and hemorrhages, sprains, allergies, frostbites, and lifesaving in and near water.

In middle school, 13–15 years, students are educated in first aid and CPR including the use of a defibrillator. Other elements that are recommended to practice are burns, diabetes, chest pain, stroke, and cramps [6, 7].

The education in lifesaving in and near water is made possible because the ability to swim is included in the requirements at the age of 12 years and 15 years. To receive a passing grade in sport and health at 12 years of age, a student must be able to swim 200 m. At the age of 15, the student must also be able to handle emergency situations by the water using alternative aids in different seasons [7].

In high school, 16–18 years, first aid and CPR are performed in some courses but not all. Some schools cooperate with emergency services or other organizations in education and training of students [7].

Although children and adolescents are trained in emergency skills and CPR, it is mainly the adults who witness out-of-hospital cardiac arrest [8]. One way to increase adults’ knowledge in CPR is to encourage children and adolescents to spread their CPR knowledge at home. By describing what they have learned and by showing educational material to their parents, the children also get the chance to consolidate their knowledge [9, 10]. In the longer term, the number of individuals being able to perform CPR increases in the society, which leads to an increase in the possibility of resuscitation and the survival rate of out-of-hospital cardiac arrests [1].

Studies related to youth CPR usually use theoretical instruments to assess CPR performance, although the assessment of practical CPR quality can be regarded as a golden standard and may need to be implemented in further evaluations. This study aimed to investigate the effect of a 2-h CPR intervention for youth.

## Methods

This study had a quantitative approach. Data was collected through an intervention utilizing simulation. The data collection consisted of a pre- and post-assessment of the participants’ CPR performance.

### Participants

Participants in this study consisted of 50 individuals, 30 females and 20 males with age between 16 and 20 years (mean 17). None of the participants had performed CPR on a human. Inclusion criteria were participation in a CPR education intervention. All participants received oral information about the study and were then asked to voluntarily participate. All asked accepted to participate.

### Intervention and data collection

The study was carried out in three steps. *Step 1* was to assess the participants’ CPR performance. Participants performed CPR for 2 min on a Brayden Pro (Innosonian®). Each of the participants performed chest compressions and ventilations according to the ERC guidelines [2]. The mechanical spring simulating the thoracic resistance required 30 kg chest compressions to achieve correct compression depth of 5–6 cm. Total recoil of the chest and the hand placement on the lower part of sternum generated 100% rating. Correct compression rate was between 100 and 120 per minute, and to achieve correct ventilation, the mouth-to-mouth ventilation required 400–700 ml per breath [2]. Data were collected on compression rate, depth, recoil, hand placement, and ventilation volume. In *step 2*, the group participated in 2-h-long education that included theory regarding anatomy and pathophysiology and hands-on training performing CPR. The intervention was completed with *step 3*. Step 3 was to once more assess the participants’ CPR performance for 2 min. No feedback about the CPR performances was given during step 1 neither step 3 assessments. All results were read from a Samsung Galaxy with associated Brayden application.

### Data analysis

The descriptive and analytic analysis was conducted using IBM Statistical Package for the Social Sciences (SPSS) 24.0. Descriptive analysis (central tendency and distribution) was used to describe the data. Analytic statistics (*t* test) were used to compare the pre- and post-assessment. The level of significance used was set at α = 0.05.

## Results

The result shows that on average, the group has improved from pre- to post-test in compression depth performing within the ERC limits (5.11 cm) (Table 1).

**Table 1:** Mean and range of compressions and ventilations divided into pre- and post-test

Item	Pre-test	Under perform	Over perform	Post-test	Under perform	Over perform	Sig. (2-tailed)
Compression depth (cm)	4.88	46%	4%	5.11	39%	6%	.064
Standard deviation	.68			.54			
Range	3–6			4–6			
Compression rate (per min)	117	15%	54%	128	6%	75%	.001
Standard deviation	17.1			14.8			
Range	70–159			92–166			
Compression recoil (%)	89	11%	0%	92	7%	0%	.404
Standard deviation	24.3			17.4			
Range	3–100			19–100			
Hand position (%)	84	16%	0%	93	7%	0%	.024
Standard deviation	23.9			13.9			
Range	8–100			29–100			
Ventilation volume (ml)	112	88%	12%	579	49%	47%	.000
Standard deviation	309.3			588.7			
Range	0–1100			0–1400			

Compression rate went from 117 in pre-test which is within ERC limits to 128 in post-test which is above the recommended rate, which is a deterioration. However, regarding the ranking, the minimum rate in the pre-test at 70 compressions has increased to minimum 92 compressions in the post-test which is an improvement.

**Fig. 1:**
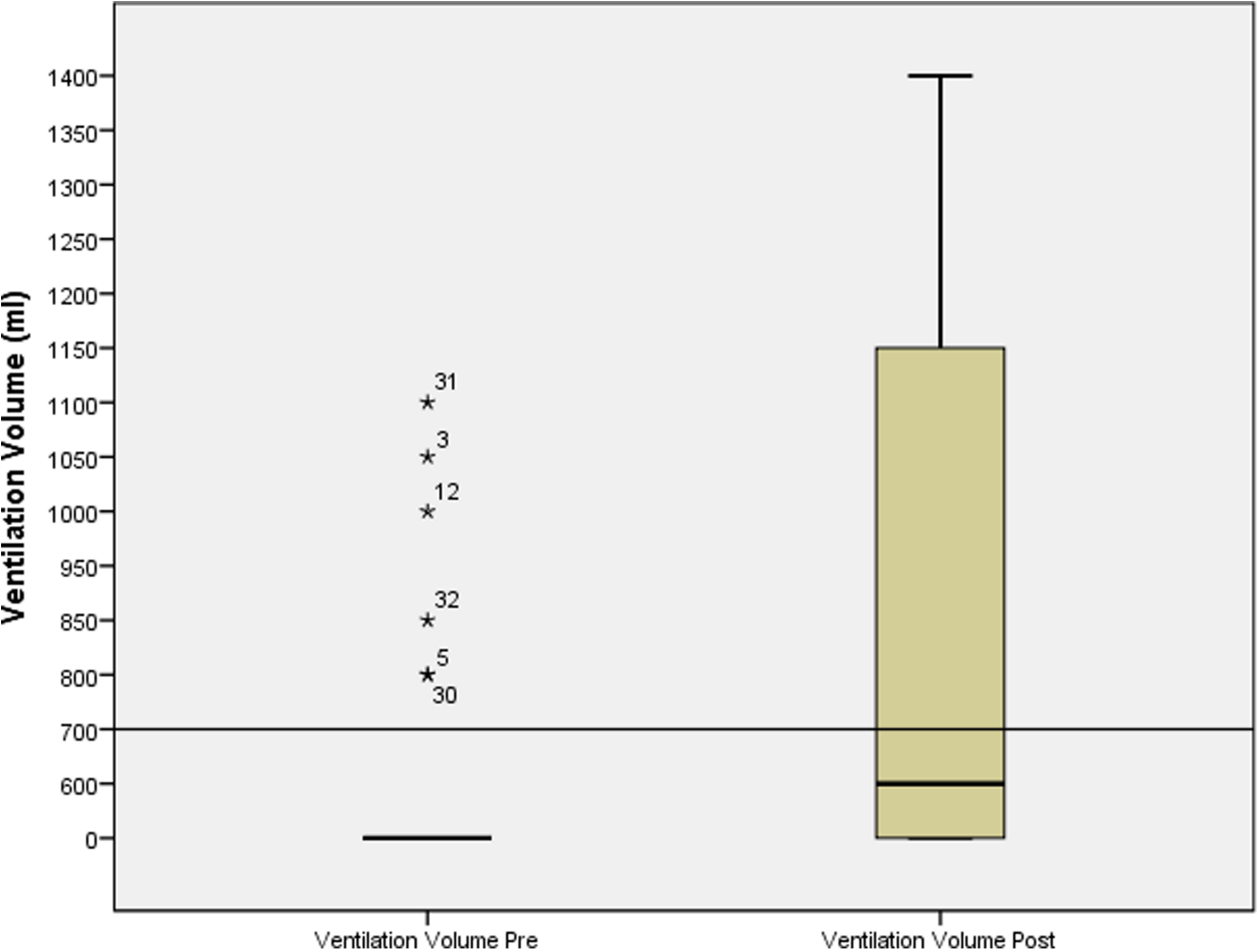
Ventilation volume in milliliters, divided in pre- and post-groups

The ventilation volume increased from 112 ml in pre-test to 579 ml in the post-test which is within the ERC limits. The improvement was statistically significant (*p* < .00) (Fig. 1).

Hand position improved by 9%. The minimum range increased from 8% in pre-test to 29% in the post-test.

No significant result was shown regarding compression recoil. Compression recoil marginally improved by a mean of 3% from pre- to post-test.

## Discussion

In the result, the post-test regarding the compression depth is performed within the ERC limit of 5–6 cm. The pre-test was at 4.88 cm which can be considered similar to the post-test at 5.11 cm. The range in post-test has reduced to be between 4 and 6 cm which is an improvement. Previous research shows that the age, in other words, weight, and length of the individual correlate with the compression depths [11–13]. Young women may find it harder to achieve proper compression depth if they have a smaller body mass index than young men. At the age of 16–18 years, Mpotos and Iserbyt [14] state that young women achieve the same test results as men of the same age. Semeraro et al. [12] report the opposite, significantly deeper compression depth by males at the age of 15–17 years compared to same-aged females. There is also research showing that young women using the 15:2 ratio accomplish better compression depth in comparison to the 30:2 ratio [15]. According to the 2015 CPR guidelines, chest compressions are supposed to be 5–6 cm of depth at a rate of 100–120 compressions per minute [16]. It is therefore not acceptable for younger age groups to make superficial chest compressions. The compression depth of younger individuals can instead be controlled by objective feedback, to ensure given quality is reached when the compressions are too shallow significantly decreasing the chance of survival [17].

Compression rate from the pre-test has increased to be above the recommended rate in the post-test. This is an apparent deterioration of CPR quality. The chest compressions are correlated to the perfusion, and therefore, chest compressions with a constant rate are to be prioritized [18]. Interrupted chest compression during CPR reduces coronary perfusion pressure [19]. But in the opposite, a compression rate at 128 per minute (mean) results in a stretching of the sterner with a negative influence on the blood volume return to the heart [18]. The minimum rate in the post-test has increased from 70 to 90 compressions per minute, which is an improvement but still below the limit. When the compression rate is too slow, the chance of survival decreases significantly [17].

This study’s result showed how the ventilation volume increased from a mean of 112 ml in the pre-test to a mean of 579 ml per ventilation in the post-test. The improvement was statistically significant. The result shows that only 6 participants in the pre-test manage to ventilate the patient while the remaining 44 participants failed with the ventilation and did not manage to get air in the patient’s lungs. This result of few participants successfully ventilating the patient has been previously demonstrated [20]. One of the reasons for this may be participants not being able to successfully establish a free airway which then means that no air gets into the lungs of the patient. There is a risk that the air instead goes down into the ventricle of the patient [21]. Another reason why the patient is not ventilated may be the high psychological barrier of youth to initiate mouth-to-mouth ventilation as described by Berthelot et al. [22]. The participants who did not want to ventilate the patient, using mouth to mouth, did not make a serious attempt to ventilate mouth to mouth.

After the intervention, the number of ventilations is divided into 47% overperforming and 49% underperforming. Overperforming results in a hyperventilation of the patient which may have a harmful effect. It contributes to poor outcomes from cardiac arrest because it decreases cardiac output due to an increase in intrathoracic pressure [23]. Cerebral vasoconstriction can also be caused by hyperventilation based on the decrease of carbon dioxide pressures in the blood, which decreases cerebral blood flow [24]. Real-time tidal volume feedback is an option in which to enhance the results in this kind of education since it has proved to be a tool for the rescuers to provide optimal ventilation. Learning with feedback means that the helper avoids hyperventilation during the CPR [25].

There is, despite the training, still 49% of the individuals underperforming the ventilation in the post-test. During the training, the young individuals have not learned to perform adequate ventilation with tidal volumes that makes the chest visibly rise [18]. This would indicate that 2 h of training is not enough to learn to ventilate according to guidelines. Bohn et al. [20] show that with an annual recurring training, it is still difficult to perform the ventilation correctly. Low-dose, high-frequency CPR training could be a way to maintain and improve students’ skills in both ventilation and compressions [26, 27].

In summary, repeated training retains or improves skills, but the format and frequency of repeated training are yet to be fully determined [28]. Interestingly enough, in this context, Iskrzycki et al. [10] show how young individuals with parents who are also involved in learning CPR had better results for a longer time. This relates to young individuals repeating and consolidates their skills when they teach their parents.

### Limitations

This study included youth from different schools in one city. The extent to which motivation and social structure influenced the results remains unclear. This study did not bring the youths’ CPR skills to perfection. However, the training may reduce anxieties about making mistakes and therefore increase the youths’ willingness to help others.

## Conclusion

Educating young individuals at school is a way to reach out with CPR knowledge in society. In this study, the ventilation of the patient is a major weakness. To be able to create an open airway and ventilate the patient with the correct volume as well as to overcome the psychological barrier to initiate mouth-to-mouth ventilation may require more training than 2 h. The training may need to consist of repeated training sessions over the year with feedback, in order to teach young individuals the skills to perform CPR with good quality.

## Data Availability

All data generated or analyzed during this study are included in this published article.

## Abbreviations

CPR: Cardiopulmonary resuscitation
EPSF: European Patient Safety Foundation
ERC: European Resuscitation Council
ILCOR: International Liaisons Committee on Resuscitation
SPSS: Statistical Package for the Social Sciences
WFSA: World Federation of Societies of Anesthesiologists
WHO: World Health Organization
